# Understanding barriers to access services among grandparents raising grandchildren

**DOI:** 10.1093/geroni/igaf122.3312

**Published:** 2025-12-31

**Authors:** Julian Montoro-Rodriguez, Bert Hayslip, Anita Rogers, Jaia Lent, Emily Patrick, Ana Beltran

**Affiliations:** The University of North Carolina at Charlotte, Charlotte, North Carolina, United States; University of North Texas, Denton, Texas, United States; Generations United, Washington, District of Columbia, United States; Generations United, Washington, District of Columbia, United States; Generations United, Washington, District of Columbia, United States; Generations United, Washington, District of Columbia, United States

## Abstract

Grandparent caregivers face numerous barriers in accessing needed services, undermining their well-being. This is key to their adjustment in coping with the demands of caregiving. This study explored predictors of multiple dimensions of barriers to service use among135 grandparent and kinship caregivers (Mage = 56.3) living in Philadelphia. Principal components analysis yielded a 3-factor model of barriers 1) negative interactions with service providers, family stress, understanding and accessing services, 2) grandparent/grandchild physical/mental health difficulties, and 3) limited health insurance, unemployment, inadequate housing, lack of transportation). Predictors were based upon the Anderson (1995) Behavioral and Strengths-Oriented (Ronch & Goldfield, 2003) models. Hierarchical regression analyses found that 1) greater age, poorer grandparent mental health, and less personal resilience uniquely predicted (p < .02) more Factor 1 barriers. For Factor 2, greater age, poorer grandparent physical health, greater mental health impediments to caregiving, and less resilience each uniquely (p < .03) predicted greater barriers. Less income, greater mental health impediments to caregiving, and less resilience uniquely (p < .03) predicted greater Factor 3 barriers. These findings suggest that Anderson predisposing variables as well as enabling and need variables are important in understanding the determinants of barriers to service among grandparent caregivers. They also suggest that the attention to one’s physical and mental health and the ability to cope with adversity, i.e., personal resilience (Rutter, 2006), are important in allowing grandparent caregivers’ to overcome barriers to service. These findings underscore the importance of adaptive coping skills/empowerment in accessing services among grandparent caregivers.

